# Editorial: Biomechanics, Health, Disease and Rehabilitation—2nd Edition

**DOI:** 10.3390/bioengineering12020121

**Published:** 2025-01-28

**Authors:** Redha Taiar

**Affiliations:** MATIM, Université de Reims Champagne-Ardenne, 51687 Reims Cedex 2, France; redha.taiar@univ-reims.fr

## 1. Introduction

Our aim in launching this Special Issue was to identify and better understand the impacts of bioengineering data applications on our comprehension of human behavior in daily life tasks. Currently, the application of engineering technologies in patients during rehabilitation training is important, as it allows us to explore, understand and optimize biological problems. Our objective was to summarize the most important methods of improving human rehabilitation performance in relation to health sciences in all age groups, throughout patients’ lives. For this Special Issue, we encouraged the submission of papers that aimed to promote the latest research in the fields of health, quality of life improvement and sport rehabilitation and to share recommendations. Our aim was to help prevent functional decline and frailty by following a life course perspective approach, utilizing the latest research, with applications in general health, as well as those targeted toward different stages of life, enabling prevention, performance improvement and management in disease. The modeling, simulation, quantification and computing of the musculoskeletal system can allow us to quantify and improve the discriminate parameters characterizing movement in different cases, including in patients’ daily lives. Our aim was to effectively combine and coordinate research and results in order to understand and improve human responses to medicine.

This Special Issue showcases ideas, approaches, opinions and comments relating to the latest research in the fields of biomechanics, health, disease and rehabilitation. Moreover, this Special Issue also provides a forum for researchers to investigate the role of the latest research in biomechanics in enhancing performance in health, disease and rehabilitation. In this Special Issue, three original research articles are included that address these questions. We will summarize their major contributions according to their subject categories. Overall, these studies show the importance of advanced technologies when applied to humans, such as in urodynamic and thermodynamic studies. The articles all focus on the use of modern technologies in improving quality of life and related applications. Many fascinating, high-quality methodologies were developed across the articles. We will now summarize their major contributions according to their subjects.

In the first original research paper, Hui-Hsuan Lau et al. [1] retrospectively review the urodynamic data of UP patients measured pre- and post-operatively. The impact of TVM-HTX on voiding resistance and associated parameters is analyzed. In conclusion, the authors’ results reveal that TVM-HTX reduces voiding resistance and, therefore, prompts urine emission, increasing voiding efficacy and reducing the pressure gradient required for driving urine flow, thereby lessening the voiding workload in high-grade UP patients. These findings, according to the authors, could be informative for clinicians when deciding on pelvic reconstruction in UP patients.

In the second original research paper, Strahovnik et al. [2] explore the correlation between the coronal alignment of the lower limb and the relative proximal tibial rotation in both mechanical and kinematic alignment strategies. They report that the existence of this relationship could help surgeons to be better prepared to reliably position the tibial component in a more anatomically correct rotation, thus avoiding rotational mismatch in total knee arthroplasty (TKA). This study shows a correlation between the tibial varus and the external rotation of the tibia. Knees with more than 5° of tibial varus (MPTA < 85°) are highly likely to show increased external tibial rotation and a pre-operative rotational femorotibial mismatch. The use of standard tibial rotational landmarks in these cases requires the further internal rotation of the tibial component to avoid excessive post-operative rotational component mismatch.

In the third original research paper, Longo et al. [3] report on their study, through which they determined how LSA and DSA are related to shoulder kinematics and clinical outcomes after RTSA. The authors’ hypothesis was that LSA would correlate with higher internal–external rotation and shoulder elevation, while DSA would be associated with higher shoulder elevation. Moreover, the relationship between shoulder kinematics and clinical outcomes after RTSA was evaluated. The results reveal that TVM-HTX reduces voiding resistance and, therefore, prompts urine emission, increasing voiding efficacy and reducing the pressure gradient required for driving urine flow, thereby lessening the voiding workload in high-grade UP patients. These findings, according to the authors, could be informative for clinicians when deciding on pelvic reconstruction in UP patients.In conclusion, the results reveal that TVM-HTX diminished voiding resistance, and, therefore, prompted urine emission to increase the voiding efficacy and reduce the pressure gradient requisite for driving urine flow, thereby lessening the voiding workload of high-grade UP patients. Their findings could be informative for clinicians when deciding on pelvic reconstruction for UP patients.

## 2. Conclusions

This Special Issue, “Biomechanics, Health, Disease and Rehabilitation—2nd Edition”, similarly to the first edition, covers advances in human health and wellbeing. The interdisciplinary nature of these studies highlights the potential of the latest technologies in shaping the future of healthcare and quality of life through related applications in human daily life.

## 3. Top of Form

This Special Issue is highly informative, covering the latest research in specific fields and enabling readers to deepen their knowledge and understanding of the complex applications of these modern technologies and their importance in our lives today.

## References

[1] Lau H.-H., Lai C.-Y., Hsieh M.-C., Peng H.-Y., Chou D., Su T.-H., Lee J.-J., Lin T.-B. (2024). Thermodynamic Work of High-Grade Uterine Prolapse Patients Undergoing Transvaginal Mesh Repair with Total Hysterectomy. Bioengineering.

[2] Strahovnik A., Strahovnik I., Fokter S.K. (2024). Coronal Knee Alignment and Tibial Rotation in Total Knee Arthroplasty: A Prospective Cohort Study of Patients with End-Stage Osteoarthritis. Bioengineering.

[3] Longo U.G., Franceschetti E., Carnevale A., Schena E., Cozza G., Perricone G., Cardinale M.E., Papalia R. (2023). Influence of Lateralization and Distalization on Joint Function after Primary Reverse Total Shoulder Arthroplasty. Bioengineering.

